# Description of Ventricular Arrhythmia after Taking Herbal Medicines in Middle-Aged Couples

**DOI:** 10.1155/2020/6061958

**Published:** 2020-10-01

**Authors:** Mohammad Ali Zakeri, Vahid Mohammadi, Gholamreza Bazmandegan, Maryam Zakeri

**Affiliations:** ^1^Social Determinants of Health Research Center, Rafsanjan University of Medical Sciences, Rafsanjan, Iran; ^2^Non-Communicable Diseases Research Center, Rafsanjan University of Medical Sciences, Rafsanjan, Iran; ^3^Department of Cardiology, Ali-Ibn Abi-Talib Hospital, School of Medicine, Rafsanjan University of Medical Sciences, Rafsanjan, Iran; ^4^Clinical Research Development Unit, Ali-Ibn Abi-Talib Hospital, Rafsanjan University of Medical Sciences, Rafsanjan, Iran; ^5^Department of Family Medicine, Ali-Ibn Abi-Talib Hospital, School of Medicine, Rafsanjan University of Medical Sciences, Rafsanjan, Iran; ^6^Physiology-Pharmacology Research Center, Research Institute of Basic Medical Sciences, Rafsanjan University of Medical Sciences, Rafsanjan, Iran

## Abstract

Medicinal herbs and some derivatives have been used in the treatment of heart disease which is rarely responsible for ventricular arrhythmias and cardiac arrest. Ventricular tachycardia (VT) increases the risk of sudden cardiac death (SCD). However, only a few reports are available about the cardiac ventricular arrhythmia followed by taking herbal medicines. We present two patients (a couple) without a history of heart disease who referred to the hospital with ventricular arrhythmia.

## 1. Introduction

VT is caused by the activity of abnormal ventricular foci. It is mostly developed due to either acute myocardial infarction or advanced cardiomyopathy [1]. VT maybe increased the risk of SCD [2]. According to SCD reports, the prevalence of cardiac arrest (CA) is 45-83.7 per 100,000 individuals [3, 4]. A study has shown stable polymorphic VT, ventricular fibrillation, and patient death due to herbal medicines [5]. Medicinal herbs and some derivatives have been used in the treatment of heart disease, including congestive heart failure, hypertension, angina, atherosclerosis, cerebral insufficiency, venous insufficiency, and arrhythmias [6]. However, many plants and some supplements used in cardiovascular therapies have potentially serious side effects and complications that need to be evaluated through clinical experiments [7]. Many herbal medicines have not been scientifically evaluated, and some of them can cause serious toxic effects and major drug interactions [6]. We will present an interesting case of ventricular arrhythmia following the use of herbal medicines to better understand the complications, diagnosis, and treatment in the early acute stages.

## 2. Case Presentation

Patients were a middle-aged couple with a history of hypertension who referred to a public hospital with acute abdominal pain and periodic palpitation. Acute abdominal pain in both patients was due to the consumption of herbal medicines for reducing emotional stress including (a) Echium amoenum (containing saponins, flavonoids, unsaturated terpenoids, and sterols [8]), (b) Citrus aurantifolia (containing flavonoids including apigenin, hesperetin, kaempferol, nobiletin, quercetin, and rutin [9]), (c) Citrus aurantium (containing limonene, *β*-myrcene, *α*-pinene, and *β*-pinene [10]), and (d) Matricaria chamomilla L. (containing sesquiterpenes, flavonoids, coumarins, and polyacetylenes [11]). These drugs were taken arbitrarily and without a doctor's prescription. The two patients had an acute fever, numbness, sweating, dryness, tingling, and abdominal pain. They used a combination of these herbal medicines only once (for the first time) and had no previous use of it.

Ventricular arrhythmia (an automatic focus, monomorphic, and nonsustained VT) without hemodynamic effects was seen in the male and female case for 24 hours and 1 hour, respectively, after receiving the first dose of amiodarone. Ultrasound, abdominal X-ray, exercise test, and echocardiography were performed in both patients, showing normal results. The patients' electrocardiogram (ECG) was normal on the second day of hospitalization, at the time of discharge, and 3 months later. After the treatment and discharge, the patients were followed up for three months (monthly) with Holter monitoring for 24 hours. No sign of arrhythmia was observed. Follow-up was performed to test the herbal medicines used; however, we did not access them.

### 2.1. Case 1

A 64-year-old male patient with no medical or coronary heart disease history referred to a public hospital for acute abdominal pain. A specialist in the emergency department clinically examined the patient's abdominal pain, weakness, and numbness. The patient had abdominal pain, weakness, numbness, sweating, dryness, and burning mouth for 1 hour. The abdominal pain was localized to the epigastric region spread to the back and got worse with 7/10 intensity after lying down. The patient reported no change in his bowel habits, no nausea and vomiting, and no history of trauma to his chest or abdomen. He also had no history of substance or alcohol use.

**Figure 1:**
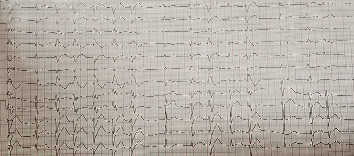
Male patient's ECG, ventricular arrhythmia (run of VT).

Abdominal pain and its symptoms appeared after drinking medicinal herbs. The abdomen was soft without tenderness and rebound tenderness. Gastrointestinal consultation showed no abnormality. The patient had a blood pressure of 80/50 and a heart rate of 115 beats per minute (bpm). The lung and heart sounds were normal and the pulse of the distal organs was full and symmetrical. The patient was also evaluated for myocardial infarction (MI), and the troponin test was negative. An irregular heartbeat was observed in the patient's ECG and cardiac monitoring (Figure 1). Ventricular arrhythmia (an automatic focus, monomorphic, variable cycle length, and nonsustained VT) with a stable hemodynamic state was diagnosed. Oxygen therapy and antiarrhythmic treatment were prescribed in the emergency department. 150 mg amiodarone was used to control the patient's arrhythmia. The patient was referred to the Coronary Care Unit(CCU) for further evaluation. He was hospitalized in the CCU for 48 hours, evaluated for any arrhythmia, and received cardiac medication. One mg/min amiodarone was used to control the patient's arrhythmia for 6 hours, and then, 0.5 mg/min amiodarone was used for 18 hours. The probabilities of cardiomyopathy as well as toxicity were rejected. Finally, the patient was discharged after 72 hours after taking the necessary examinations.

### 2.2. Case 2

A 50-year-old woman with no family history of coronary heart disease referred to a public hospital for acute abdominal pain for 1 hour. The specialist in the emergency department examined the patient. She complained of abdominal pain, fatigue, and generalized weakness without dizziness. The patient had tingling and numbness of the lower jaw and hands.

The abdominal pain was localized to the epigastric region with 6/10 intensity, which was constant in nature and did not radiate elsewhere. The patient had no recent travel and no sign of substance or alcohol use.

**Figure 2:**
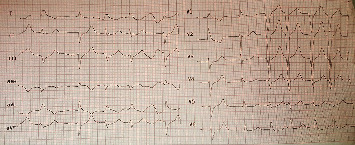
Female patient's ECG, ventricular arrhythmia (run of VT).

Abdominal pain and associated symptoms appeared after drinking medicinal herbs. The patient's abdomen was soft with no tenderness. She had a history of hypothyroidism and hysterectomy. Her blood pressure was 95/50 mmHg with a heart rate of 92 bpm. Gastrointestinal counseling showed no abnormality. The patient's ECG showed ventricular arrhythmia (monomorphic with fusion beats, an automatic focus, variable cycle length, and nonsustained VT) at a rate of 92 bpm (Figure 2). Oxygen therapy and antiarrhythmic treatment were provided in the emergency department. The patient was evaluated for MI. Troponin test results were negative for the patient. 150 mg amiodarone was used to control the patient's arrhythmia in the emergency department. She was immediately transferred to the cardiac care unit and received 1 mg/min amiodarone for 6 hours and then 0.5 mg/min for 18 hours. The probability of toxicity was rejected. Finally, the patient was discharged after 72 hours with necessary clinical examinations.

## 3. Discussion

Herbal medicines have been used for centuries over time. However, the side effects associated with them need to be evaluated and understood [12]. Two important herbal medicines used to treat heart disease are Echium amoenum and Citrus aurantium in the southern regions of Iran [13]. Studies have shown the positive role of Echium amoenum in cardiac remodeling in an animal model with MI and congestion [14]. The effect of Echium amoenum on the blood pressure and heart rate in an animal model was studied. This plant did not cause significant damages to the heart tissue. Significant changes in erythrocytes and related parameters were observed. High doses of Echium amoenum should be used with caution due to its blood toxicity [15]. However, Hamidi and Khaksari showed the effect of Echium amoenum and Citrus aurantiflia on animal blood pressure and heart rate. Echium amoenum has a hypertensive effect, so it is not recommended. Compound consumption of Echium amoenum and Citrus aurantiflia is effective in reducing heart rate [16]. These findings indicate different effects of these plants on the heart. Therefore, the hypothesis that “compound effects of herbal medicines may be different from those of the herbal medicines alone” is strengthened.

Citrus aurantium extract contains p-synephrine. It is generally assumed that p-synephrine will increase heart rate and blood pressure [17, 18]. However, Seifert et al. showed that Citrus aurantium extract did not lead to increased cardiovascular stress (hypertension and heart rate) [19]. Moreover, Stohs showed that the adverse effects of Citrus aurantium extract were unjustified [20]. However, a 22-year-old healthy man was reported to develop myocardial infarction after taking dietary supplements containing Phenorex, the main ingredient of Citrus aurantium. Although the use of these plants separately has been associated with adverse cardiac events, no sufficient information is available about the concomitant use of these plants [21]. Khouri et al. studied the cardiac effects of Citrus aurantium and showed that it inhibited the electrophysiological properties of the ventricular lobe in a concentration-dependent, time-dependent, and rapidly unrelated model. It also had inhibitory and antiarrhythmic effects when used for treating supraventricular tachyarrhythmia in animal models [22].

Jambi studied the cardiac effects of Matricaria chamomilla L. and showed the effectiveness of this extract in reducing doxorubicin-induced cardiac toxicity [23]. Furthermore, Chamomile tea can reduce the severity of short breath and anxiety in patients with chronic heart failure [24]. No further study on the cardiac effects of Matricaria chamomilla L. was found. While herbal products are mainly used for their therapeutic effects, the use of multiple medicines increases the risk of drug interactions and side effects [25]. The present study demonstrated the erythema caused by some herbal medicines. Medications have diverse effects, and several herbal medicines can be used simultaneously. However, these effects cannot be attributed to one or even all herbal medicines. Few studies have investigated and reported side effects of herbal medicines. This being so, further studies are required due to lack of scientific evidence about the safety and side effects of such medicines.

## 4. Conclusion

Since herbal medicines are widely used for treating various diseases, it is necessary to pay attention to their toxic effects on cardiac function. Health professionals should consider the use of herbal medicines and their effects on the diagnosis and treatment of patients. The evaluation of the patients showing herbal side effects can provide valuable insights to better understand clinical effects of these medicinal plants and identify adverse cardiovascular reactions.
